# Specific roles for the PI3K and the MEK–ERK pathway in IGF-1-stimulated chemotaxis, VEGF secretion and proliferation of multiple myeloma cells: study in the 5T33MM model

**DOI:** 10.1038/sj.bjc.6601613

**Published:** 2004-03-02

**Authors:** E Menu, R Kooijman, E Van Valckenborgh, K Asosingh, M Bakkus, B Van Camp, K Vanderkerken

**Affiliations:** 1 Department of Hematology and Immunology, Vrije Universiteit Brussel – VUB, 1090 Brussels, Belgium; 2 Department of Neuroendocrine Immunology, Vrije Universiteit Brussel – VUB, 1090 Brussels, Belgium; 3 Laboratorium of Molecular Hematology, AZ-VUB, 1090 Brussels, Belgium

**Keywords:** multiple myeloma, homing, signal transduction, VEGF, IGF-1

## Abstract

Insulin-like growth factor-1 (IGF-1) has been described as an important factor in proliferation, cell survival and migration of multiple myeloma (MM) cells. Angiogenesis correlates with development and prognosis of the MM disease. Vascular endothelial growth factor (VEGF) is one of the prominent factors involved in this process. The different functions of IGF-1 were investigated in the 5TMM mouse model with emphasis on proliferation, migration and VEGF secretion, and the signalling pathways involved. Western Blot analysis revealed that ERK1/2 and Akt (PKB) were activated after IGF-1 stimulation. The activation of ERK1/2 was reduced by the PI3K inhibitor Wortmannin, implying that the PI3K pathway is involved in its activation. Insulin-like growth factor-1 induced an increase in DNA synthesis in MM cells, which was mediated by a PI3K/Akt-MEK/ERK pathway. Insulin-like growth factor-1 enhanced F-actin assembly and this process was only PI3K mediated. Stimulation by IGF-1 of VEGF production was reduced by PD98059, indicating that only the MEK–ERK pathway is involved in IGF-1-stimulated VEGF production. In conclusion, IGF-1 mediates its multiple effects on MM cells through different signal transduction pathways. In the future, we can study the potential *in vivo* effects of IGF-1 inhibition on tumour growth and angiogenesis in MM.

Multiple myeloma (MM) represents a B-cell malignancy, characterised by a monoclonal proliferation of the plasma cells in the bone marrow (BM) where they secrete high levels of immunoglobulins. The MM cells induce osteolysis, by activating osteoclasts, and increase neovascularisation or angiogenesis (Vacca *et al*, 1994).

Insulin-like growth factor (IGF-1) is mainly produced in the liver (Jones and Clemmons, 1995; Le Roith *et al*, 2001), but is also produced in considerable quantities by other cells (e.g.BMstromal cells) (Ferlin *et al*, 2000). We and others have shown that IGF-1 plays an important role in MM. Although IL-6 has mostly been described as a proliferation factor for MM, it has become clear that IGF-1 has an equally important proliferative and antiapoptotic effect (Ferlin *et al*, 2000; Tu *et al*, 2000; Qiang *et al*, 2002). It could be that IGF-1 plays an even more pivotal role in the survival of MM cells as IL-6 independent lines still respond to IGF-1 (Ferlin *et al*, 2000; Qiang *et al*, 2002). Our group has demonstrated that IGF-1 serves as a chemoattractant for MM cells (Vanderkerken *et al*, 1999). As we have reported that MM cells have a postgerminal origin (Bakkus *et al*, 1992), this implies that the cells then need to enter or re-enter the BM where they interact with the BM stromal cells and receive proliferation and survival signals (Caligaris-Cappio *et al*, 1991; Uchiyama *et al*, 1993; Van Riet and Van Camp, 1993; Lokhorst *et al*, 1994). This migration from the vascular to the extravascular compartment of the BM is called ‘homing’. Butcher and Picker (1996) described the process of homing of lymphocytes as a multistep event in which chemoattractants are essential to attract and activate the lymphocytes.

Angiogenesis is a relatively new factor that plays a role in MM development. The vascular endothelial growth factor (VEGF) has been shown to be expressed in human MM (Vacca *et al*, 1999). Our group has recently reported that the VEGF isoforms 120 and 164 are expressed in the 5TMM mouse model (Van Valckenborgh *et al*, 2002). Vascular endothelial growth factor has a vital role in angiogenesis, as it is able to stimulate vascular permeability and is an endothelial cell-specific mitogen (Connolly *et al*, 1989).

In this work, the role of IGF-1 in the 5T33MM model was investigated, with special emphasis on the ability of IGF-1 to stimulate proliferation, migration and VEGF secretion. The signal transduction pathways that are activated in response to IGF-1 were determined. We studied the two most well known, distinct downstream pathways of IGF-1 namely the PI3K and the MAPK pathway (Bornfeldt *et al*, 1994; Puglianiello *et al*, 2000; Tu *et al*, 2000; Qiang *et al*, 2002). The 5TMM model was used since this is an ideal *in vivo* model for testing different inhibitors of IGF-1. The 5TMM cell lines originated spontaneously in aging C57BL/KaLwRij mice and have since been propagated *in vivo* by intravenous transfer of the tumour cells in young syngeneic mice. This model is representative for the human disease; the tumour cells have a predominant localisation in the bone marrow, normal Ig concentration is decreased and is associated with an increased serum paraprotein that is correlated with the development of the disease. The presence of the tumour cells in the BM is associated with enhanced angiogenesis (Van Valckenborgh *et al*, 2002) and induction of bone lesions. On a more cellular basis, a similar profile of adhesion molecules and chemokine receptors is observed and similar mechanisms are used to induce osteoclastogenesis (Radl *et al*, 1979; Vanderkerken *et al*, 1997; Vanderkerken *et al*, 2000; Asosingh *et al*, 2000a).

We here demonstrate for the first time that IGF-1 stimulates VEGF secretion by MM cells through activation of ERK. Proliferation of the MM cells in response to IGF-1 was mediated through a PI3K–ERK pathway, while migration was mediated through PI3K. These *in vitro* data demonstrate the importance of studies whereby IGF-1 function is inhibited *in vivo*. The 5TMM model hereby offers a suitable model combining these *in vitro* data with *in vivo* work.

## MATERIALS AND METHODS

### Animals

C57BL/KaLwRij mice were purchased from Harlan CPB (Horst, The Netherlands). Mice were used when they were 6–10 weeks old. They were housed and treated following the conditions approved by the Ethical Committee for Animal Experiments, VUB (license no. LA1230281). The animal ethics meet the standards required by the UKCCCR Guidelines (UKCCCR, 1998).

### MM models

The 5T33MM cells originated spontaneously in aging C57BL/KaLwRij mice and have since been propagated by intravenous transfer of the diseased marrow in young syngeneic mice. The paraprotein was assessed by protein electrophoresis of the serum samples. When the serum concentration reached 10 mg ml^−1^, the mice were killed and the BM was flushed out of the femurs and tibiae and crushed out of the vertebrae. The BM cells were suspended in serum-free medium (RPMI 1640 (GIBCO, Life Technologies, Ghent, Belgium), supplemented with penicillin–streptomycin, glutamine and MEM NEAA-pyruvate (GIBCO)). The cells were then purified by Lympholyte M (Cedarlane, Hornby, Canada) gradient centrifugation at 1000 **g** for 20 min. After washing, the cells were further purified by centrifugation for 25 min at 450 **g** on a 70% iso-osmotic Percoll (Pharmacia, Uppsala, Sweden) gradient. The cell band on top of the gradient contained enriched 5T33MM cells, with a purity reaching 90–95%, as measured by flow cytometric analysis. Viability was more than 95%.

### Stimulations and inhibitions

The cells were kept in serum-free medium and were stimulated with 100 ng ml^−1^ recombinant human IGF-1 (animal/media grade, Grow Pep Ltd, Adelaide, Australia), for all experiments except for the thymidine incorporation assays where it was 10 ng ml^−1^. The inhibitors Wortmannin (Sigma, Irvine, UK), Ly294002 (Sigma), PD98059 (Alexis, CA) and UO126 (Alexis) were added 30 min prior to IGF-1 stimulation at a concentration of 100 nM, 10 *μ*M, 20 *μ*M and 25 *μ*M, respectively. The inhibitors were dissolved in DMSO. The DMSO concentration was 0.001% and the same concentration was used as vehicle. This DMSO concentration was tested on the MM cells and no change in function was measured compared to untreated cells.

### Western blot analysis

The cells were first starved in serum-free medium for 1 h to reduce increased basal levels of ERK 1 and 2 and Akt before stimulating them. After stimulation with IGF-1, the cell pellets were lysed in lysis buffer containing 50 mM Tris, 150 mM NaCl, 1% NP40 and 0.25% sodium deoxycholate. The following protease and phosphatase inhibitors were added: 4 mM Na_3_VO_4_ (Sigma), 1 mM Na_4_P_2_O_7_ (Sigma), 50 mM NaF (VWR, PA, USA), 5 mM EDTA (VWR), 1 mM AEBSF (ICN, CA, USA), 2 *μ*g ml^−1^ aprotinin (Sigma), 50 *μ*g ml^−1^ leupeptin (Sigma), 50 *μ*g ml^−1^ pepstatin A (ICN), 500 *μ*g ml^−1^ trypsin inhibitor (Sigma), 10 *μ*M benzamidin (Sigma) and 2.5 mM pnp benzoate (Sigma). The cells were then cleared by centrifugation (5 min, 13 000 **g**) and sample buffer was added. After boiling, the samples were separated on a 10% SDS–PAGE and transferred to PVDF membranes (Bio Rad, CA, USA). The membranes were blocked with PBS containing 5% low fat milk and 0.1% Tween 20 and probed with the appropriate antibodies, namely anti-pThr^202^/Tyr^204^ ERK, anti-pThr^180^/Tyr^182^p38 and anti-pThr^183^/Tyr ^185^JNK. For measuring total protein levels, the blots were stripped and reprobed with pan Ab. Anti-p44/42MAPK, anti-p-T202/Y204 p44/42MAPK, anti-Akt and the HRP-coupled secondary antibodies anti-rabbit and anti-mouse were acquired from Westburg, MA, USA, while anti-p-S473Akt was acquired from Biosource, CA, USA. The bands were visualised using the ECL system (Amersham, Buckinghamshire, UK).

### Thymidine incorporation assays

Cells (1.10^6^ ml ^−1^) were incubated in serum-free medium with or without IGF-1 for 17 h, in the presence or absence of the inhibitors. At 16 h before harvesting, cells were pulsed with 1 *μ*Ci (methyl-^3^H) thymidine (Amersham). Cells were harvested by a cell harvester (Inotech, Wohlen, Switzerland) on paper filters (Filtermat A, Wallac, Turku, Finland). Filters were dried for 1 h in a 60°C oven and sealed in sample bags (Wallac) containing 4 ml Optiscint Scintillation Liquid (Wallac). Radioactivity was counted using a 1450 Microbeta Liquid Scintillation Counter (Wallac). Results are expressed as the relative DNA synthesis: the amount of radioactivity of the unstimulated cells was set to 1 and the fold increase of the radioactivity (c.p.m.) of the stimulated cells compared to this is shown.

### Quantifying F-actin content

The MM cells were kept in serum-free medium and were stimulated with IGF-1 for 10 min. The cells were then fixed for 15 min with 3% paraformaldehyde in PBS. Cells were washed with PBS and quenched in 0.1 M glycine in PBS for 15 min. After a second wash, the cells were permeabilized with 0.2% Triton X-100 in PBS containing 1% bovine serum albumin (BSA, Boehringer Manheim, SA, Germany) for 10 min. The cells were then treated with 0.5 *μ*M FITC-conjugated phalloidin (Molecular Probes, OR, USA) for 30 min. All the incubations were performed at room temperature. After washing, the cells were analysed by flow cytometric analysis (FACScalibur, Becton Dickinson, LA, USA). These results are normalised similar to those of the thymidine incorporation assay.

### Vascular endothelial growth factor ELISA

Vascular endothelial growth factor ELISAs were performed according to the manufacturer's instructions (Quantikine M, mouse VEGF ELISA, R&D systems, MN, USA). In short, MM cells were kept in serum-free RPMI 1640 medium at a density of 1.10^6^ cells ml^−1^ with or without IGF-1 for 24 or 48 h. Conditioned media of the MM cells were brought on a plate coated with anti-VEGF. After 2 h, the plate was washed and an antibody against VEGF conjugated to HRP was added. After another 2 h, the plate was washed and the substrate solution was added for 30 min. Then the reaction was stopped and the fluorescence was read on an ELISA reader (Thermo Max, Molecular Devices, CA, USA) at 450 nm with a correction set to 540 nm. The results were then plotted against the standard curve to become the actual concentrations. These results are normalized similar to those of the thymidine incorporation assay.

### RNA isolation and cDNA synthesis

Total RNA from 5 × 10^6^ cells was isolated using the SV total RNA isolation system (Promega, Madison, WI, USA) according to the manufacturer's instructions. The concentration and purity of RNA were determined by spectrophotometric measurement (Gene Quant II: Pharmacia Biotech, Cambridge, UK). The total RNA was converted into cDNA by the superscript first-strand synthesis system (GIBCO) using random hexamers as primers.

### Real-time quantitative PCR

In real-time quantitative PCR, a dual-labelled fluorogenic probe that contains a fluorescent reporter dye at the 5′ end and a quencher dye at the 3′ end is used. When the probe is intact, emission from the reporter dye is quenched by the quencher. During the extension phase of PCR, the annealed probe is cleaved by the 5′ nuclease activity of *Taq* polymerase, releasing the reporter from the quencher. This results in an increase of fluorescence emission from the reporter dye, which can be quantitatively detected by the ABI (Foster City, CA, USA) PRISM 7700 Sequence Detector. The amount of fluorescence measured in a sample is proportional to the amount of specific PCR product generated. By analysis of the data, the *C*_t_ value can be determined. This parameter represents the PCR cycle at which an increase in fluorescence is detectable above the baseline signal.

In the 5TMM model, only the VEGF 120 and 164 isoforms can be detected (Van Valckenborgh *et al*, 2002). Primers and probe for the detection of these isoforms were found in the article of Zhang *et al* (2002) and purchased from Eurogentech (Seraing, Belgium). Both isoforms share the common probe, 5′-ACA GCA GAT GTG AAT GCA GAC CAA AGA AAG-3′ and the common forward primer, 5′-GCC AGC ACA TAG AGA GAA TGA GC-3′. The VEGF 120 reverse primer was 5′-CGG CTT GTC ACA TTT TCT GG-3′, while the VEGF 164 reverse was 5′-CAA GGC TCA CAG TGA TTT TCT GG-3′. Primers were located in two different exons to eliminate detection of genomic DNA. The endogenous reference gene GAPDH was used to standardise the amount of sample RNA. Primers and probes for GAPDH were purchased from ABI and used according to the manufacturer's instructions. The GAPDH and VEGF probes were labelled with a quencher dye (TAMRA) and a reporter dye (FAM for VEGF and VIC for GAPDH). Taqman PCR was performed in a 25 *μ*l reaction mix containing 12.5 *μ*l 2 × master mix (ABI), 200 nM probe, 300 nM forward primer, 300 nM reverse primer and 50 ng cDNA for the detection of VEGF mRNA. The PCR cycles consisted of an initial denaturation step at 95°C for 10 min followed by 40 cycles at 95°C for 15 s and 60°C for 1 min. Each sample was amplified in triplicate. The relative standard curve method was used to quantitate the relative VEGF expression in the 5T33MM cells treated with IGF-1. Relative standard curves (50, 10, 2, 0.4, 0.083 ng) were prepared using cDNA from 5T33MM control samples.

### Data analysis real-time PCR

According to the manufacturer's instructions, the relative standard curve method was used. Standard curves were generated by plotting *C*_t_ values against the log input amount of RNA. The input amount of VEGF and GAPDH of unknown samples was calculated with the following formula: log input amount=(*C*_t_ value−*b*)/*m*, where *b* is the *y* intercept of the standard curve line and *m* is the slope. Next, the amount of VEGF mRNA was divided by the amount of GAPDH mRNA to determine the normalised amount of VEGF mRNA. The value of the control sample was then set to 1 so that the values of the IGF-1-treated samples would be expressed as a fold increase.

### Statistical analysis

For statistical analysis, the Mann–Whitney test was used. *P*⩽0.05 was considered significant.

## RESULTS

### Activation of signal transduction elements in response to IGF-1 stimulation

**Figure 1:**
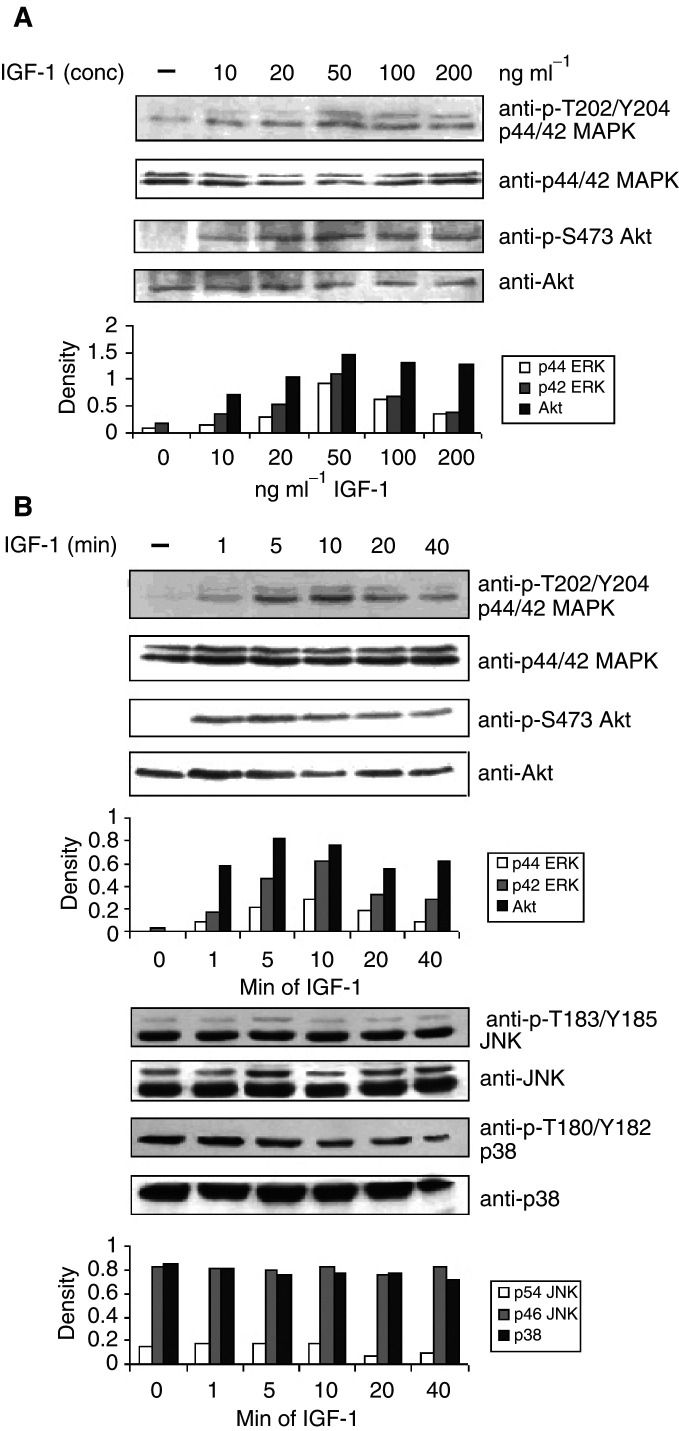
Activation of the MEK–ERK and the PI3K pathway after IGF-1 stimulation. (**A**) 5T33MM cells were stimulated with increasing concentrations of IGF-1 for 10 min. (**B**) 5T33MM cells were stimulated during different periods of time with 100 ng ml^−1^ IGF-1. Equivalent amounts of lysates were immunoblotted with anti-P-ERK1/2 (first panel), P-Akt (third panel), P-Jnk (fifth panel) and P-p38 (seventh panel) and reblotted with anti-ERK1/2 (second panel), anti-Akt (fourth panel), anti-JNK (sixth panel) and anti-p38 (eigth panel) to confirm equal loading. One experiment representative of four is shown.

We have already shown by FACS analysis that the 5T33MM cells express the IGF-1R (Asosingh *et al*, 2000b). As a first step in defining the IGF-1 signalling pathways, the phosphorylation by IGF-1 of the MAP kinases ERK1/2, p38 and JNK was determined. The cells were stimulated with different concentrations of IGF-1 (Figure 1a

) and for different time periods (Figure 1b). The MAPK p38 and JNK were already phosphorylated in unstimulated cells, and IGF-1 did not influence their phosphorylation state (Figure 1b). ERK1/2 on the other hand became phosphorylated after 5 min stimulation with 100 ng ml^−1^ IGF-1 and this activation continued for at least 40 min (Figure 1b).

**Figure 2:**
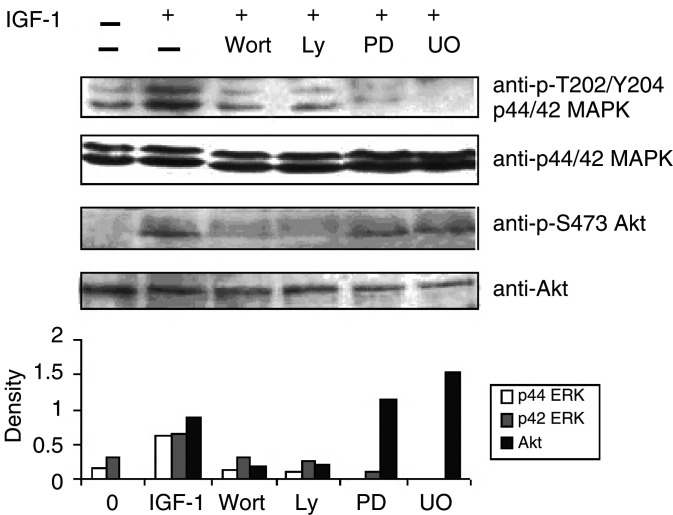
Crosstalk between the PI3K pathway and the MEK–ERK pathway. The MEK inhibitors PD98059 and UO126 abolish stimulation by IGF-1 of ERK phosphoryation but have no influence on the phosphorylation of Akt (first and third panel). The inhibitors of the PI3K pathway Wortmannin and Ly294002, on the other hand, inhibit the phosphorylation of Akt (PKB), confirming that Akt becomes phosphorylated through activation of the PI3K pathway (third panel), but also reduces the phosphorylation of ERK1 and 2 (first panel). The cells were stimulated with 100 ng ml^−1^ IGF-1 for 10 min and lysates were treated as in the previous figure. One experiment representative of four is shown.

To study the effect of IGF-1 on the PI3K pathway, the phosphorylation of Akt (PKB) was investigated. Akt is a downstream target of PI3K-generated signals and becomes activated after phosphorylation of Ser 473. In response to IGF-1 stimulation, Akt became phosphorylated after 1 min and an increased level of phosphorylation was observed for at least 40 min (Figure 1b). Since Akt phosphorylation is completely inhibited with PI3K inhibitors, Akt is a useful molecule to measure PI3K activation (Figure 2

).

Qiang *et al* (2002) recently described a crosstalk between the MAPK pathway and the PI3K pathway in MM cells. To confirm this, the PI3K inhibitors Ly294002 and Wortmannin and the MEK inhibitors PD98059 and UO126 were used. As shown in Figure 2, the MEK inhibitors only influenced the activation levels of ERK1/2. The PI3K inhibitors on the other hand not only abolished the phosphorylation of Akt but also abrogated the activation of ERK1/2. Insulin-like growth factor-1 thus activates ERK1/2 through activation of MEK via a PI3K signalling pathway. This hypothesis is in accordance with our finding that stimulation of Akt phosphorylation takes place before the increase in ERK phosphorylation (Figure 1b).

### Signal transduction in proliferation

Figure 3

**Figure 3:**
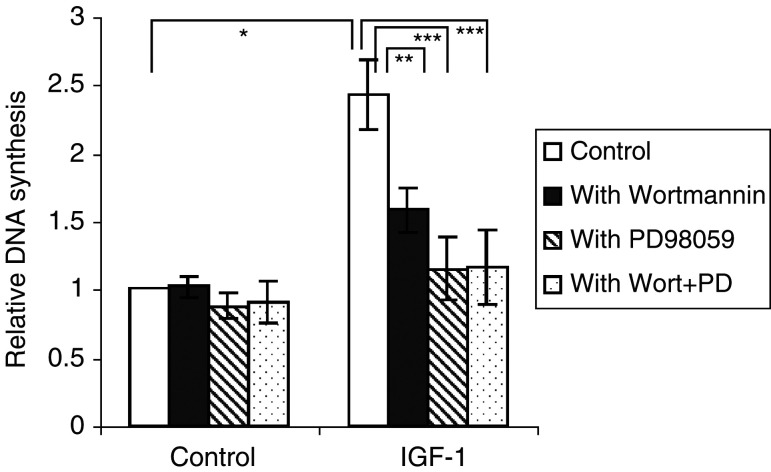
IGF-1 induced DNA synthesis. For the thymidine incorporation assays, the MM cells were incubated with RPMI in the absence or the presence of 10 ng ml^−1^ IGF-1. Before stimulation with or without IGF-1, the cells were preincubated for 30 min with Wortmannin (100 nM), PD98059 (20 *μ*M) or both where indicated. Mean values±s.d. for four independent experiments are shown. (^*^: *P*<0.01 *vs* control, ^**^: *P*<0.01 *vs* IGF-1, ^***^: *P*<0.01 *vs* IGF-1).

shows that IGF-1 augments DNA synthesis by 100%. Antiapoptotic effects of IGF-1 were measured with annexin V-PI staining; IGF-1 induced only a 1–5% decrease in apoptosis and cell death (data not shown). To address the signalling pathways involved, the cells were preincubated with inhibitors of MEK or PI3K. It appeared that Wortmannin and PD98059 reduced the effect of IGF-1 by 60 and 90%, respectively. When PD98059 and Wortmannin were added together, no additive effect was seen (Figure 3). Both inhibitors had only a minor effect on the DNA synthesis of unstimulated cells. As depicted in Figure 2, the PI3K pathway influences the phosphorylation status of the ERKs. Probably, the PI3K–MEK–ERK pathway mediates the DNA synthesis of the MM cells in response to IGF-1.

### Signal transduction in F-actin assembly

**Figure 4:**
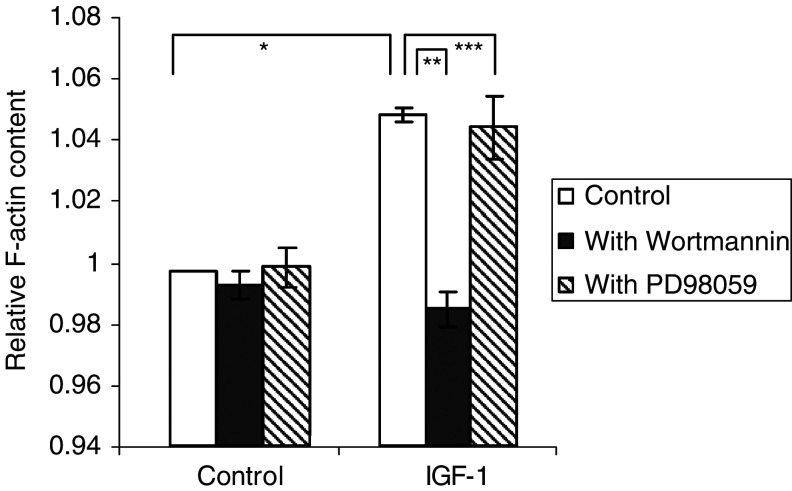
IGF-1 induced F-actin assembly. The F-actin content of the MM cells was measured by FACS analysis. The mean fluorescence intensity is shown as the relative value compared to unstimulated cells. The cells were stimulated with or without 100ng ml^−1^ IGF-1, after a 30 min incubation with Wortmannin (100 nM) or PD98059 (20 *μ*M) where indicated. The cells were then labelled with phalloidin FITC. Mean values±s.d. for three independent experiments are shown (^*^: *P*<0.02 *vs* control, ^**^: *P*<0.04, ^***^: *P*>0.05 *vs* IGF-1).

Our group has already described the chemotactic effect of IGF-1 on the 5TMM cells (Vanderkerken *et al*, 1999; Asosingh *et al*, 2000b). We have previously shown that F-actin assembly correlates with MM cell migration (Menu *et al*, 2002). Therefore, in this study, F-actin quantification was used as a measure of migration. Here we studied, through F-actin quantification, the IGF-1 signalling pathway that leads to F-actin assembly. Insulin-like growth factor-1 significantly augmented the F-actin quantity and this effect was completely abolished by Wortmannin, which had no effect in the absence of IGF-1. PD98059 had no significant effect (Figure 4

), indicating that F-actin assembly in MM cells in response to IGF-1 is mediated through the PI3K pathway and not through the MEK–ERK cascade.

### Signal transduction in VEGF secretion

**Figure 5:**
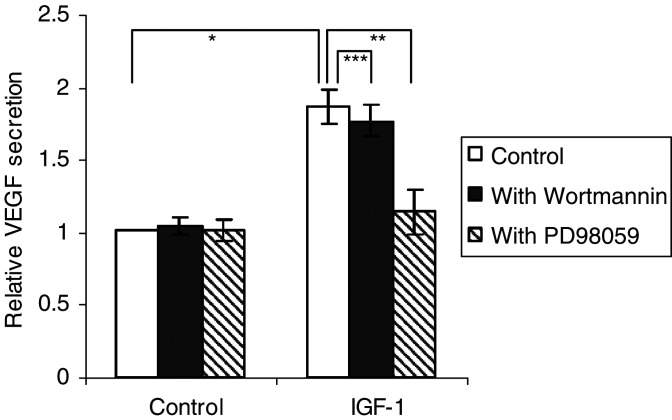
Stimulation by IGF-1 of VEGF secretion. The 5T33MM cells were stimulated with or without 100 ng ml^−1^ IGF-1 for 24 h, after a 30 min incubation with Wortmannin (100 nM) or PD98059 (20 *μ*M) where indicated. Concentrations of VEGF are shown relative to unstimulated cells. The maximum IGF-1-stimulated VEGF secretion reaches 250pg ml^−1^. Mean values±s.d. for three independent experiments are shown (^*^: *P*<0.01 *vs* control, ^**^: *P*<0.01, ^***^: *P*>0.05 *vs* 100 ng ml^−1^ IGF-1).

**Figure 6:**
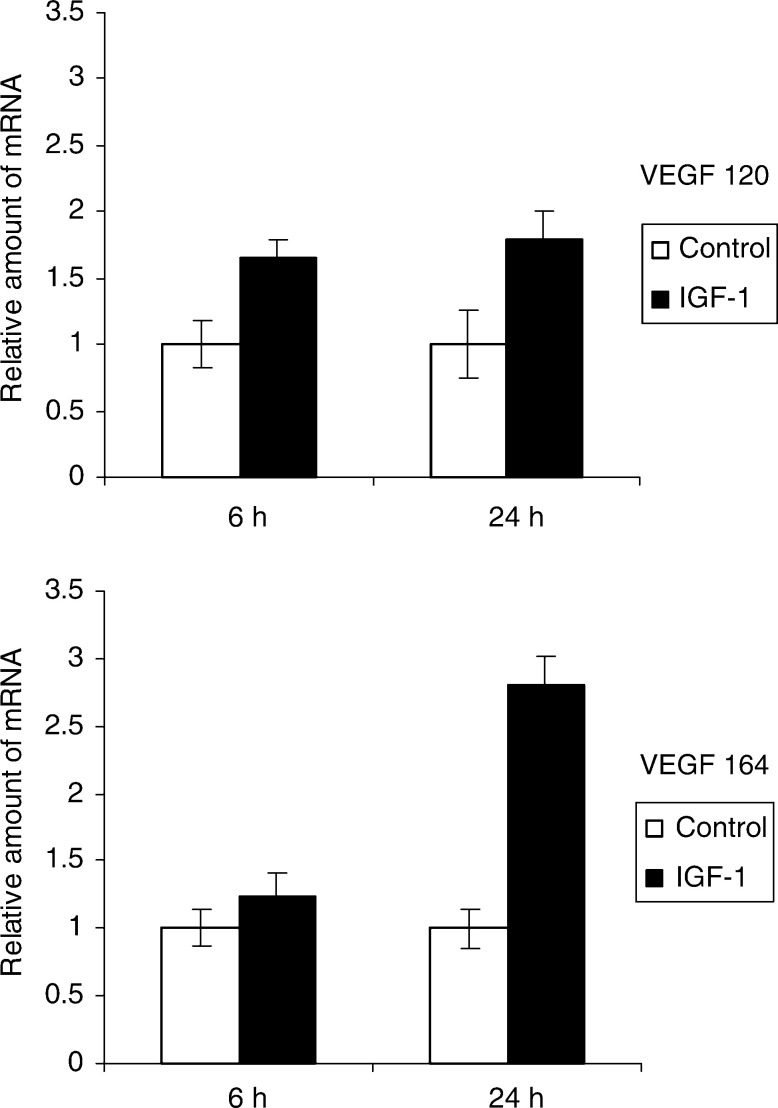
IGF-1 stimulates VEGF mRNA expression. The MM cells were stimulated with 100 ng ml^−1^ IGF-1 for 6 or 24 h. The relative amount of VEGF in the IGF-1-stimulated samples compared to the control samples is shown. Error bars represent s.d. in the experiment. One experiment representing three is illustrated, *P*<0.05.

We previously described that the 5TMM cells produce VEGF (Van Valckenborgh *et al*, 2002). Figure 5

shows that IGF-1 increases VEGF levels in the conditioned media of MM cells by 100% (up to 250 pg ml^−1^) within 24 h. Flow cytometric analysis revealed that there was only a 10–15% increase in cell number in response to IGF-1 (data not shown), indicating that proliferation is not responsible for the 100% increase in VEGF concentration. The antiapoptotic effect of IGF-1 was also measured here through annexin V-PI staining. There was only 1–5% decrease in apoptosis and cell death in the IGF-1-stimulated group in this time frame (results not illustrated). This result excludes the possibility that IGF-1 increases VEGF secretion through inhibition of apoptosis. Therefore, we conclude that IGF-1 stimulates VEGF secretion in the 5T33MM cells. To determine whether the increase in production of VEGF was regulated at the mRNA level, quantitative real-time PCR was performed. In the 5TMM model, only the VEGF 120 and 164 isoforms are present (Van Valckenborgh *et al*, 2002). Figure 6

shows that after 24 h, the expression of the VEGF120 isoform was upregulated 1.8 times and that of VEGF164 2.8 times. These results suggest that VEGF production by IGF-1 is, at least in part, regulated at the mRNA level.

Next, the pathways through which IGF-1 stimulates VEGF secretion were examined. The MEK inhibitor PD98059 reduced the stimulation of VEGF production by approximately 75%, whereas the PI3K inhibitor, Wortmannin, had no significant effect (Figure 5). Both inhibitors had a minor or no effect on the unstimulated VEGF production. These results suggest that VEGF secretion by MM cells in response to IGF-1 is mediated by the MEK–ERK pathway.

## DISCUSSION

In spite of extensive research and the development of novel therapies, MM remains a deadly malignancy. One of the main characteristics of the disease is the restricted localisation of the malignant plasma cells in the BM. This is most likely due to selective homing and survival in the BM. ‘Homing’ is the process of migration from the vascular to the extravascular compartment of an organ. Butcher and Picker (1996) have described the homing process as a multistep event. Our group has already demonstrated the selective homing of the MM cells to the BM (Vanderkerken *et al*, 2000). Once in the BM, the MM cells interact with the stromal cells to receive proliferation and survival signals, unique to the BM. Vacca *et al* (1994) have also stated that angiogenesis is involved in MM and correlates with MM progression.

Insulin-like growth factor-1 is produced by the BM stromal cells (Abboud *et al*, 1991; Ferlin *et al*, 2000) and has been implicated in the development of MM. Our group has demonstrated that it acts as a chemoattractant for MM cells (Vanderkerken *et al*, 1999), and thus plays a role in the process of homing. Insulin-like growth factor-1 also stimulates the proliferation and survival of MM cells (Freund *et al*, 1994; Georgii-Hemming *et al*, 1996; Ferlin *et al*, 2000; Qiang *et al*, 2002) and has been described to increase the expression of VEGF and thus angiogenesis in colon cancer cells (Fukuda *et al*, 2002). In the present paper, we studied the *in vitro* effects of IGF-1 on 5T33MM and the involvement of the different signal transduction pathways in the stimulation of VEGF production, proliferation and F-actin assembly. As mentioned previously, the 5T33MM model is a suitable model representative for the human disease, and allowing the combination of both *in vitro* and *in vivo* experiments.

When IGF-1 binds to its receptor, it activates the tyrosine kinase activity of the receptor leading to phosphorylation of tyrosine residues on the receptor and IRS proteins. These tyrosine residues serve as docking sites for SH2 domains on other signalling molecules such as SHC or the p85 subunit of PI3K. Recruitment of SHC to the IGF-1 receptor leads to activation of the MEK–ERK pathway through activation of ras and raf (Jones and Clemmons, 1995; Le Roith *et al*, 2001). ERK is mostly known to be involved in proliferation (Ferlin *et al*, 2000; Qiang *et al*, 2002), although several groups have stated that in some cell types it could also be involved in migration (Bonacchi *et al*, 2001; Delehedde *et al*, 2001; Tanimura *et al*, 2002). The PI3K/Akt pathway is said to be an antiapoptotic pathway in myeloma (Tu *et al*, 2000) although numerous groups have found that PI3K plays a key role in migration and gradient-sensing in other cell types (Gerszten *et al*, 2001; Comer and Parent, 2002; Curnock *et al*, 2002; Stephens *et al*, 2002; Weiner, 2002).

Our results show that the MEK–ERK pathway and the PI3K/Akt pathway are activated in 5T33MM cells upon IGF-1 stimulation. Activation of the PI3K pathway occurred before activation of the MEK–ERK pathway. The other MAP kinases p38 and JNK were already activated in unstimulated cells but their phosphorylation status did not change after stimulation with IGF-1. We also observed that treatment of the cells with the PI3K inhibitors Wortmannin and Ly294002 led to inhibition of IGF-1-stimulated ERK1/2 phosphorylation, indicating a regulatory role for the PI3K pathway in the activation of ERK1/2. Qiang *et al* (2002) have also seen this crosstalk in human myeloma cells. They showed that the phosphorylation of MEK1/2 was also inhibited but not that of Raf, an upstream kinase of MEK1/2, suggesting that the effect was at the level of MEK1/2. In contrast, two groups reported that Akt could influence the phosphorylation of Raf in other cell types, thereby regulating the MEK–ERK pathway (Wennstrom and Downward, 1999; Zimmermann and Moelling, 1999). Since Akt is activated sooner than ERK1/2 and the PI3K inhibitors can diminish ERK phosphorylation, we propose a model of ERK activation through the PI3K/Akt pathway in MM cells stimulated with IGF-1.

Our observation that inhibition of the MEK–ERK pathway as well as inhibition of the PI3K pathway reduces the stimulation of DNA synthesis by IGF-1 is in accordance with our hypothesis that ERK is activated through stimulation of the PI3K pathway. Although Qiang *et al* (2002) also showed that activation of ERK in human MM cells is mediated by the PI3K pathway, they found that only the PI3K pathway was involved in myeloma cell proliferation. Our observation that the MEK inhibitor completely blocks the stimulating effect of IGF-I on proliferation indicates that the activation of ERK is essential for stimulation of 5T33MM cell proliferation. The inhibitive effect of Wortmannin in our experiments can obviously be explained by inhibition of ERK activation. However, this does not exclude an additional role for the PI3K pathway through stimulation of Akt in the stimulatory effect of IGF-I on 5T33MM cell proliferation.

We have already described the migration of the 5TMM cells towards IGF-1, but to our knowledge, no other group working on MM has described the signal transduction involved. We have already demonstrated by microscopy, fluometry and flow cytometry that polarisation of MM cells and thus F-actin assembly correlates with migration (Menu *et al*, 2002). Although the increase in mean fluorescence intensity is rather small, it correlates well with the polarisation seen by microscopy. When the F-actin assembly is blocked, migration becomes impaired (Menu *et al*, 2002). Our finding that induction of F-actin assembly by IGF-1 was blocked by the PI3K inhibitor but not by the MAPK inhibitor is in line with most papers on the involvement of PI3K in chemotaxis and migration. It has been proposed that PI3K regulates cell migration through activation of Pyk2 and FAK (Reiske *et al*, 1999; Rumsey *et al*, 2001; Franitza *et al*, 2002). Insulin-like growth factor-1 thus attracts MM cells through a PI3K signalling mechanism.

Although the importance of VEGF in MM has been described, no group has shown that IGF-1 can induce VEGF secretion in MM cells. We recently showed that the VEGF 120 and 164 isoform are the only ones produced in the 5TMM mouse model (Van Valckenborgh *et al*, 2002). Real-time Quantitative PCR revealed that mRNA of both isoforms were upregulated in MM cells stimulated with IGF-1. The secretion of the protein correlated with the mRNA upregulation. Using MAPK and PI3K inhibitors, it was found that the IGF-1-stimulated VEGF production is mediated by the MEK/ERK pathway and not by PI3K since Wortmannin had no significant effect on VEGF secretion. We postulate that it is possible that activation of ERK occurs through another pathway (e.g. the ras–raf pathway), later than the PI3K-dependent one. This is in line with observations from other groups (Miele *et al*, 2000; Kijowski *et al*, 2001; Fukuda *et al*, 2002) showing that IGF-1-induced VEGF expression in colon cancer cells and fibroblasts occurs through the MEK/ERK pathway. The observation that IGF-1 can trigger VEGF production is a novel finding and of importance. We can assume that when IGF-1 stimulates VEGF secretion by the MM cells in the BM, this will induce endothelial cell growth and vascularisation. As we have already shown that the IGF-1R expression on MM cells is upregulated after contact of the MM cells with BM endothelial cells (Asosingh *et al*, 2000b), this enhanced vascularisation will increase the effect of IGF-1 even further creating a repeating circle. Moreover, BM endothelial cells can also support the proliferation of MM cells in a way similar to fibroblasts (Szelenyi *et al*, 2002). Podar *et al* (2001, 2002) have described that VEGF on itself can also act as a migration and proliferation factor for MM cells.

It has been reported that IGF-1 upregulates MMP-9 in breast cancer cells (Mira *et al*, 1999). This matrix metalloproteinase is involved in the invasion of the MM cells through the BM matrix. We investigated whether IGF-1 can also upregulate MMP9 in our model by gelatin zymography. However, IGF-1 did not affect MMP9 secretion (data not shown).

In summary, MM cells are attracted to the BM by different factors. Insulin-like growth factor-1 is identified as an *in vitro* chemoattractant involved in this process and induces a clear polarisation of the MM cells and a rearrangement of their F-actin. Once the MM cells are in the vascular compartment, IGF-1 can stimulate their growth and increases VEGF production. The latter in turn stimulates their proliferation even more and enhances the effect of IGF-1 through upregulation of the IGF-1R after contact with the EC.

We have shown here that in the 5T33MM model, IGF-1 activates similar functions and signalling pathways as in human cells. Although difference in the usage of these pathways have been found (e.g. the use of ERK activation for stimulation of proliferation in 5T33MM cells), the 5T33MM model still allows us to investigate the *in vivo* effects of specific inhibitors of the signalling pathways or receptor tyrosine kinase inhibitors to inhibit IGF-1 function *in vivo* and in this way study the potential effects of IGF-1 inhibition on tumour growth and angiogenesis in MM.
